# Alternative splicing enriched cDNA libraries identify breast cancer-associated transcripts

**DOI:** 10.1186/1471-2164-11-S5-S4

**Published:** 2010-12-22

**Authors:** Elisa N Ferreira, Maria CR Rangel, Pedro F Galante, Jorge E de Souza, Gustavo C Molina, Sandro J de Souza, Dirce M Carraro

**Affiliations:** 1 Laboratory of Genomics and Molecular Biology, Hospital A.C. Camargo, Fundação Antonio Prudente, São Paulo, 01509-900, Brazil; 2 Department of Genetics and Evolutionary Biology, Institute of Biosciences University of São Paulo, São Paulo, 05508-090, Brazil; 3 Laboratory of Computational Biology, Ludwig Institute for Cancer Research, São Paulo, 01323-903, Brazil

## Abstract

**Background:**

Alternative splicing (AS) is a central mechanism in the generation of genomic complexity and is a major contributor to transcriptome and proteome diversity. Alterations of the splicing process can lead to deregulation of crucial cellular processes and have been associated with a large spectrum of human diseases. Cancer-associated transcripts are potential molecular markers and may contribute to the development of more accurate diagnostic and prognostic methods and also serve as therapeutic targets. Alternative splicing-enriched cDNA libraries have been used to explore the variability generated by alternative splicing. In this study, by combining the use of trapping heteroduplexes and RNA amplification, we developed a powerful approach that enables transcriptome-wide exploration of the AS repertoire for identifying AS variants associated with breast tumor cells modulated by *ERBB2* (*HER-2/neu*) oncogene expression.

**Results:**

The human breast cell line (C5.2) and a pool of 5 ERBB2 over-expressing breast tumor samples were used independently for the construction of two AS-enriched libraries. In total, 2,048 partial cDNA sequences were obtained, revealing 214 alternative splicing sequence-enriched tags (ASSETs). A subset with 79 multiple exon ASSETs was compared to public databases and reported 138 different AS events. A high success rate of RT-PCR validation (94.5%) was obtained, and 2 novel AS events were identified. The influence of *ERBB2*-mediated expression on AS regulation was evaluated by capillary electrophoresis and probe-ligation approaches in two mammary cell lines (Hb4a and C5.2) expressing different levels of *ERBB2*. The relative expression balance between AS variants from 3 genes was differentially modulated by *ERBB2* in this model system.

**Conclusions:**

In this study, we presented a method for exploring AS from any RNA source in a transcriptome-wide format, which can be directly easily adapted to next generation sequencers. We identified AS transcripts that were differently modulated by *ERBB2*-mediated expression and that can be tested as molecular markers for breast cancer. Such a methodology will be useful for completely deciphering the cancer cell transcriptome diversity resulting from AS and for finding more precise molecular markers.

## Background

More than 30 years ago, Gilbert predicted the existence of protein variants due to the alternative use of exon-intron borders in eukaryotic cells [1]. This prediction has been continually confirmed as a common feature of many species, including humans. Recent estimations, based on high-throughput sequencing, suggest that 90-95% of multiple-exon human genes undergo alternative splicing (AS) [2,3], producing an average of six distinct transcripts from each gene [4]. This phenomenon enormously impacts the repertoire of proteins, since 80% of AS events occur within the coding region [5], thus interfering in the functional aspects of the cells.

AS regulates important processes, such as embryonic development, cellular differentiation and apoptosis, by the generation of different protein isoforms among distinct tissues, developmental stages and pathological conditions [6-8]. Alterations of the splicing process, such as the loss of expression balance between variants and aberrant splicing, can lead to the deregulation of crucial cellular processes and are consequently associated with a large spectrum of human diseases [9], including cancer [10-12].

The development of methodologies to explore transcriptome diversity resulting from AS has been shown to be a potent tool, not only for improving the biological basis of cancer but also for searching for more precise molecular markers for diagnostic, prognostic and therapeutic purposes [13,14]. Different strategies for large-scale AS variant exploration have been used with different goals. Sequence and microarray-based approaches have been used for defining the AS repertoire of human cells. The former includes several computational analyses concerning genomic and transcriptome alignments of human ESTs (expressed sequence tags) and mRNA databases [11,15-17] and cross-species alignment from closely related organisms [18,19]; the latter includes genomic and exon-intron junction microarray platforms [20-23]. Both approaches have contributed to the investigation of the expression pattern of AS variants and also facilitated the identification of novel AS variants. Nonetheless, both approaches are impaired in detecting low-abundance AS transcripts. In this sense, AS-enriched cDNA libraries is one of the most interesting approaches because it combines the convenience of cDNA direct sequencing with the advantage of detecting low-abundance transcript variants. The methodology is based on one enrichment step, consisting of the trapping of heteroduplex molecules formed by the hybridization of two distinct AS variants from the same gene [24]. The heteroduplex can be captured by molecules that recognize the heteroduplex structure [25,26], generating a vast number of AS events without previous knowledge of them. In this study, to explore AS variants associated with breast tumor cells, we established a powerful approach that enabled the direct exploration of an AS repertoire by combining the use of trapping heteroduplex and RNA amplification. To favor the trapping of splicing variants associated with breast tumor cells that over-expresses the *ERBB2* (*HER-2/neu*) oncogene, a human breast cell line (C5.2) and a pool of 5 ERBB2 over-expressing breast tumor samples were used. Two AS-enriched libraries were constructed, generating a set of 2,048 partial cDNA sequences, named here as alternative splicing sequence-enriched tags (ASSETs), as suggested by Watahiki and collaborators [25]. A subset with 79 ASSETS representing distinct multiple exon sequences was explored in this analysis and reported 138 different AS events. A high rate of validation by RT-PCR (94.5%) was obtained, and 2 novel AS events were identified. Moreover, the balance in the expression level of the AS variants from 3 genes was influenced by *ERBB2*-mediated expression.

The approach presented here certainly will contribute to the identification of the AS repertoire of cancer cells, especially as it is potentially applicable to any cell type from any tumor tissue, since a small amount of total RNA is required with no previous cDNA library construction. Furthermore, it is completely suitable for using with next-generation sequencers, substantially increasing its potential in deciphering the AS diversity in cancer cell transcriptome.

## Results

### Alternative splicing libraries

Two distinct AS libraries were constructed (Lib_1 and Lib_2) using 5 µg of total RNA as the starting material. Library 1 (Lib_1) was prepared from the human breast cell line C5.2, which over-expresses the oncogene *ERBB2*, and library 2 (Lib_2) was prepared from a pool of 5 invasive breast carcinoma samples that stained positively for ERBB2 according to immunohistochemistry analysis (Table 1).

**Table 1:** Clinical characteristics from the ductal carcinoma samples.

Sample Name	Stage	Age	TNM	LN	Grade	Molecular Markers
**9T**	IIa	55 years	T2N0M0	Negative	Grade I SBR	ER +/ PR +/ p53 -/ ERBB2+ (3+)
**20T**	IIb	87 years	T2N0M0	Negative	Grade II SBR	ER +/ PR -/ p53 -/ ERBB2+ (3+)
**22T**	IIb	56 years	T2N1M0	Positive	Grade III SBR	ER +/ PR -/ p53 -/ ERBB2+ (2+/3+)
**28T**	IIIa	42 years	T2N2M0	Positive	Grade II SBR	ER +/ PR -/ p53 -/ ERBB2+ (3+)
**36T**	I	45 years	T1N0M0	Negative	Grade III SBR	ER +/ PR -/ p53 -/ ERBB2+ (3+)

Age: age of diagnosis; TNM: classification according to TNM (T – size; N – lymph node status; M – presence of metastasis); LN: involvement of sentinel lymph nodes; grade: grades I, II and III according to SBR; molecular markers: ER – estrogen receptor; PR – progesterone receptor; p53 – protein TP53; and ERBB2 – protein ERBB2.

**Figure 1:**
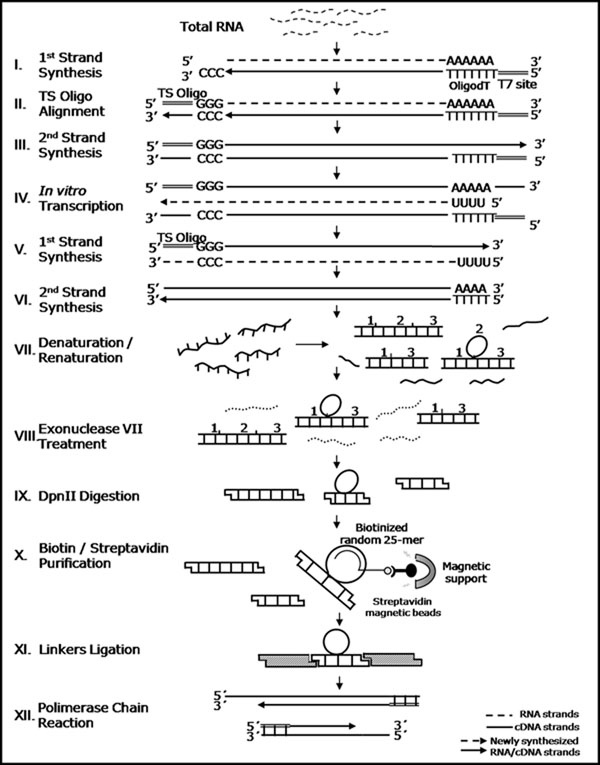
**Schematic view of the alternative splicing library construction with amplification of RNA.** I. Oligo dT containing T7 RNA Polymerase recognition site was used for first strand cDNA synthesis with Superscript II that adds cytosine residues after reaching the 5`end of mRNAs. II. This c-rich region serves as anchor for TS-oligo alignment, allowing further polymerization to the end of the oligo. III. Second strand cDNA synthesis using TS-oligo. IV. Amplification of mRNA using T7 RNA Polymerase. V. First strand cDNA synthesis using TS-oligo. VI. Second strand cDNA synthesis using oligodT. VII. Denaturation and renaturation resulting in the formation of heteroduplexes molecules by common exons complementarity. VIII. Single-stranded molecules degraded by Exonuclease (dotted line). IX. DpnII digestion resulting in small cohesive fragments. X. 25mer biotinilated random oligos coupled to streptavidin magnetic beads anneal to single-strand loops. XI. Coupling of specific adaptors to the cohesive ends of the captured heteroduplexes. XII. PCR amplification of fragments using adaptors specific oligos (double line).

The strategy for AS library construction was based on the methodology described by Watahiki and collaborators [25] with some modifications. One significant difference was the use of total RNA instead of parental full-length cDNA libraries, which simplifies the process and decreases costs. Another important adaptation was the inclusion of a RNA amplification procedure based on T7 RNA polymerase and Template Switch oligo (TS-oligo) [27], which allows the use of small quantities of RNA (Figure 1 I-IV). The amplified RNA was converted into double-stranded cDNA (dscDNA) (Figure 1 V-VI), which was then submitted to denaturation and renaturation steps, promoting the formation of heteroduplex DNA molecules by the hybridization of complementary regions from two distinct splicing variants from the same gene (Figure 1 VII). The remaining single-stranded molecules or overhanging regions were removed with exonuclease VII treatment (Figure 1 VIII), whereas the double-stranded cDNA molecules were cleaved with the *Dpn*II restriction enzyme (Figure 1 IX). This step resulted in double-stranded fragments, constituting homo- and heteroduplex molecules with cohesive ends to bind adaptors. The enrichment of the heteroduplex molecules occurred through the trapping of single-stranded loops by the annealing of random 25-mer biotinilated oligonucleotides captured by streptavidin magnetic particles (Figure 1 X). Complementary overhanging adaptors were then specifically ligated to the cohesive ends of the heteroduplex molecules (Figure 1 XI), generating a recognition site for primer annealing and consequently allowing for PCR amplification (Figure 1 XII), cloning and sequencing.

**Figure 2:**
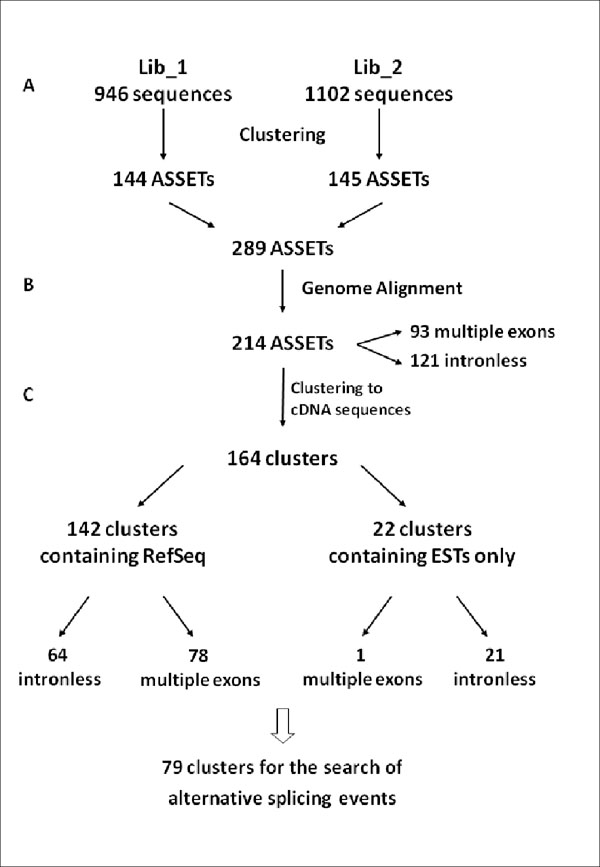
Flowchart of the bioinformatics pipeline.

A total of 2,048 high quality sequences (Phrep > 20) were generated from both libraries. Sequences from each library were clustered using the CAP3 program [28], resulting in 149 consensus sequences for library Lib_1 (96 contigs and 53 singlets) and 146 consensus sequences for library Lib_2 (74 contigs and 74 singlets) (Table 2 and Figure 2A). The number of consensus sequences obtained revealed, as expected, a high redundancy within the libraries (Table 2), since no normalization procedure was implemented in our approach.

**Table 2:** Characterization of libraries Lib_1 and Lib_2

Library	# High Quality Sequences	# Contigs	# Singlets	# Consensus	Redundancy
Lib_1	946	96	48	144	84.78%
Lib_2	1102	74	71	145	86.84%
Total	2048	170	119	289	-

All consensus sequences were then aligned to the human genome (NCBI build #36.1) using BLAST [29] and Sim4 [30], where only the best hit was considered. Based on criteria for identity (≥ 93%) and coverage (≥ 55%), 214 consensus sequences were aligned on the human genome, 93 and 121 of them reporting multiple and one-exon(s) sequences, respectively (Figure 2B). The consensuses were termed ASSETs, as previously proposed [25,26].

**Figure 3:**
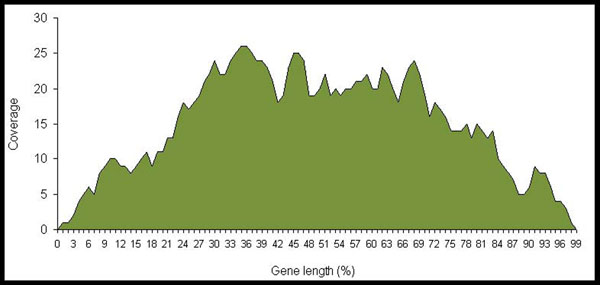
**Relative position of the ASSETs throughout full-length mRNAs.** The graphic represents the distribution of the ASSETs along corresponding transcript position. In the x-axis the relative transcript position is shown as a percentage value, where 0 indicates the 5’ end and 100 indicates the 3’ end. The coverage is the number of ASSETs aligning at each position.

Furthermore, to check whether our library construction approach enables full-length representation, including the 5’ end of transcripts, we verified the relative position of the ASSETs throughout the length of full-lengths (Figure 3). The analysis resulted in a similar representation of 3’ and 5’ ends with a slightly higher concentration of ASSETs in the central region, indicating that no bias were introduced towards higher representation of full transcript 3’ ends. Additionally, the fact that the average size of mRNAs (RefSeq) represented by ASSETs in our libraries was of 2,836 nt, similar to the average size of all mRNA sequences from RefSeq database (3,098 nt) suggested no bias in representation of 5’ end from short transcripts (Figure 3).

### Detection of alternative splicing events

No distinct splicing variants were observed among the sequences belonging to the same consensuses that would be indicative of putative AS events. Therefore, we searched for AS events through comparisons between ASSETs and full-length or partial cDNA sequences available in public databases.

First, ASSETs were clustered with ESTs from dbEST (8,133,299 ESTs), mRNAs (244,284 sequences) and RefSeqs (26,040 sequences) downloaded from UCSC (September 2007) (Figure 2C). This step resulted in 164 clusters, where 142 contained at least one RefSeq sequence. Sixteen clusters contained sequences from both libraries (Lib_1 and Lib_2), revealing an overlap of approximately 10%.

The 79 clusters containing ASSETs with multiple exons were scanned for AS events through pairwise comparisons of exon/intron boundaries between the ASSET and the reference sequences of each cluster. AS events were searched within the region delimited by the two outermost overlapping regions of each ASSET related cluster. For each ASSET, the corresponding gene and the number and type of related alternative splicing events were annotated.

All 79 multiple exon ASSETs were considered known transcripts since they were represented by sequences at public databases. Moreover, for 39 out of 79 ASSETs (49.4%), an alternatively spliced transcript was described in the public databases. For these 39 ASSETs, 138 AS events were detected, including intron retention (5.8%), exon skipping (9.4%), alternative splice site 3’ (39.8%) and alternative splice site 5’ (44.9%). The remaining 40 ASSETs, to which no AS event has been reported, may result from novel AS events not yet reported in public databases (Table 3). The intronless ASSETs were not used for the AS search, since it is not possible to identify the direction of transcripts in the absence of splice sites. Nonetheless, it is interesting to note that 63 out of 96 intronless sequences (65.6%) aligned to regions involved in AS, according to public databases. This can be considered an indirect sign that these ASSETS are prone AS transcripts.

**Table 3:** Search for AS variants by comparison with sequences from public databases.

	Presence of alternatively spliced transcripts in databases	No alternatively spliced transcripts in databases
**Lib_1**	ATP1A1*	*CDC42SE1*
	*ATP5A1*	CDK5RAP2*
	*C6orf108*	*DDB2*
	*CAMK2G*	*EEF2*
	*CD320*	*FARS2*
	*CTSH*	*GABARAP*
	*ELF3*	*GNB3*
	FLNA*	*GRK6*
	*GAPDH*	*HDAC2*
	*GNAS*	ITGB5*
	*GNPTAB*	*MAN2A1*
	*MAN1B1*	*OSBPL8*
	*NAP1L1*	*PSMD6*
	*PPIB*	*PTPRA*
	*RANBP1*	*188268*
	*RPL28*	RBM10*
	*RPL6*	*RNF149*
	*RPS4X*	*ROCK2*
	*SETD2*	*RPL11*
	SFRS9*	*THSD1*
	*STK25*
	*UQCRC1*
**Lib_2**	ALDH3A2*	*ACLY*
	AOF2*	*ASCC3L1*
	*CCNB1*	*C7orf55*
	*CREB3*	COL7A1*
	*DNAJC10*	*DDEF1*
	FN1*	*DENND4C*
	*INTS9*	*GDF9*
	*MYO1C*	*KIAA0090*
	RPS2*	*KIAA0152*
	*RPS5*	*MRPL45*
	*SEC61G*	*PHF19*
	*ST13*	PTPLA*
		*RBMX*
		*SGSM2*
		*SLC4A2*
		TRIP6*
		*XPO1*
**Lib_1 & Lib_2**	CLTC*	*ATXN10*
	EIF4A3*	*INPP1*
	GSPT1*	*PAIP1*
	KRT18*	
	PSMC2*	

*ASSETs selected for RT-PCR validation

### Gene ontology annotation

**Figure 4:**
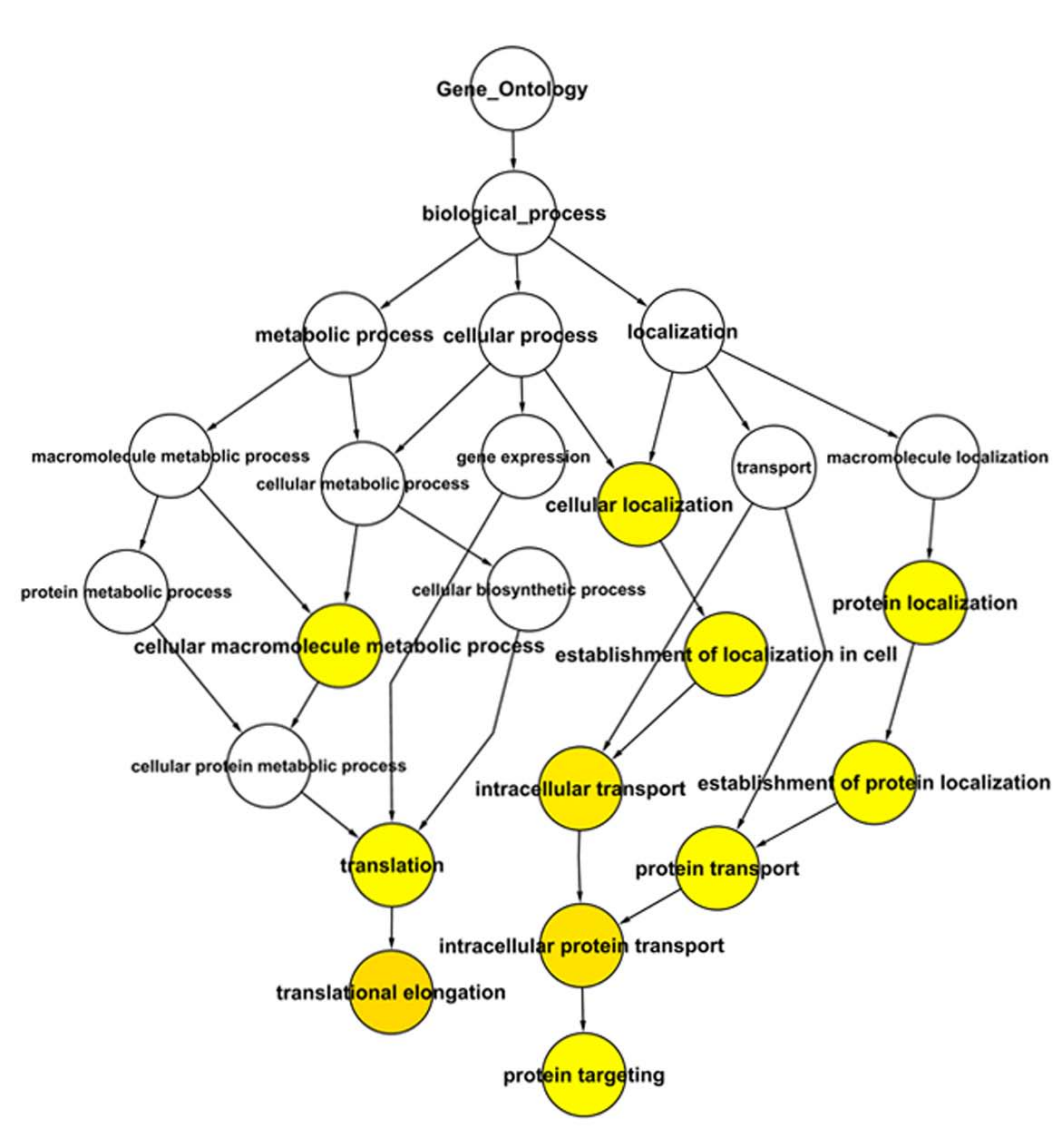
**Graphical view of GO Biological Process overrepresented categories.** The graphic is represented in a hierarchical form. The yellow circles correspond to the categories that were statistically significantly enriched.

For exploring the functional aspects of the genes that harbor AS, the 142 ASSETs were classified within the biological process categories. Using BinGO tools [31], 11 categories revealed a statistically significant enrichment of genes (Table 4) and are represented in a hierarchical form in Figure 4. The most significantly enriched category was translation elongation, due to a great number of ribosomal proteins detected in our data.

**Table 4:** Functional classification of genes within the statistically significant biological process categories.

GO-ID Description	Corrected p value	Gene Symbol
**Translation Elongation**	1.67E+01	*RPL6 RPL21 EEF2 RPL11 RPS4X RPS2 RPS5 RPL28*
**Intracellular Protein Transport**	4.21E+01	*XPO1 CLTC GABARAP KRT18 YWHAH NUP62 ZFYVE16 KPNA6 RPL11 MRPL45 SEC61G SEC61A1 SRP9*
**Intracellular Transport**	7.66E+01	*XPO1 MYO1C CLTC GABARAP YWHAH KRT18 NUP62 ZFYVE16 SEC22B KPNA6 RPL11 RANBP1 GNAS MRPL45 SRP9 SEC61G SEC61A1*
**Cellular Localization**	2.55E+02	*XPO1 MYO1C VIL2 CLTC GABARAP YWHAH KRT18 NUP62 ZFYVE16 SEC22B KPNA6 GNAS RPL11 RANBP1 MRPL45 SRP9 SEC61G SEC61A1*
**Establishment of Localization in Cell**	3.06E+02	*XPO1 MYO1C CLTC GABARAP YWHAH KRT18 NUP62 ZFYVE16 SEC22B KPNA6 RPL11 RANBP1 GNAS MRPL45 SRP9 SEC61G SEC61A1*
**Cellular Macromolecule Metabolic Process**	3.81E+02	*PPP6C XPO1 UQCRC1 CAMK2G PTPLAD1 FARS2 DNAJC10 MAN1B1 RPS2 RPL6 PTPLA RPL11 PSMD6 DNAJA3 GLT25D1 STK25 ROCK2 PAIP1 PTPRA ZDHHC7 AXL MOBKL1A EEF2 RPS4X RPS5 RPL28 IFNAR1 CCNB1 MGAT1 ST13 SENP1 HDAC2 GSPT1 PPIB RPL21 PSMC2 DDB2 GRK6 MRPL45 CTSH*
**Protein Targeting**	3.81E+02	*XPO1 ZFYVE16 KPNA6 RPL11 GABARAP SRP9 SEC61G*
**Protein Localization**	3.81E+02	*XPO1 VIL2 CLTC GABARAP YWHAH KRT18 NUP62 ZFYVE16 SEC22B KPNA6 RPL11 GNAS MRPL45 SEC61G SEC61A1 SRP9*
**Translation Elongation**	3.81E+02	*GSPT1 RPL6 RPL21 PAIP1 FARS2 EEF2 RPL11 RPS4X RPS2 MRPL45 RPS5 RPL28*
**Protein Transport**	3.81E+02	*XPO1 CLTC GABARAP YWHAH KRT18 NUP62 ZFYVE16 KPNA6 SEC22B RPL11 GNAS MRPL45 SEC61G SEC61A1 SRP9*
**Establishment of Protein Localization**	3.81E+02	*XPO1 CLTC GABARAP YWHAH KRT18 NUP62 ZFYVE16 KPNA6 SEC22B RPL11 GNAS MRPL45 SEC61G SEC61A1 SRP9*

### Validation of ASSETs and heteroduplexes

Eighteen ASSETs were randomly selected for RT-PCR validation, including 6 and 7 ASSETs exclusively from Lib_1 and Lib_2, respectively and 5 ASSETs that were detected in both libraries. The validation process was performed in two steps: *i.* ASSET validation - to confirm the presence of the ASSET in the same RNA used for library construction and *ii.* heteroduplex validation - to search for alternatively spliced transcripts that could have participated in the heteroduplex formation (Table 5). By using a pair of primers that aligned at the extremities of the ASSET sequence, all but one ASSET was validated (17 out of 18, 94.4% validation rate). The 5 ASSETs identified by both libraries were validated in both templates. Secondly, for 6 (*SFRS9*, *FLNA*, *ALDH3A2*, *PTPLA*, *RPS2* and *TRIP6*) out of the 17 validated ASSETs (35.3%), an additional AS variant was identified that could have participated in the heteroduplex formation. Two out of 6 AS variants that were transcribed from the genes *PTPLA* and *TRIP6,* which were not described in public databases, are novel splicing variants. The lack of heteroduplex validation for the other 11 genes was probably due to a differential expression balance between splicing variants that precluded the amplification of one variant in favor of the most abundant one. The support for this assumption is that for 5 out of 11 (45.5%) genes, an AS variant that could have participated in the heteroduplex formation was available in databases. For verifying whether the low level of overlap between both libraries was due to the low coverage in terms of the number of sequences generated for each library or due to the specific AS pattern of each RNA source used, we tested if the 13 ASSETs validated in cDNA from the corresponding library were also expressed in the cDNA from the other library. Four ASSETs from the 5 identified by Lib_1 were successfully amplified using the cDNA from the pool of the tumor samples (Lib_2). All 7 ASSETs from Lib_2 were successfully amplified using the cDNA from C5.2 (Lib_1), totaling 91.8% cross-validation (11 out of 12). The validation results are summarized in Table 5.

**Table 5:** RT-PCR validation.

Library Origin	Selected ASSETs	ASSET validation	Heteroduplex validation	Cross-validation
**Lib_1**	6	5	2	4
**Lib_2**	7	7	4	7
**Lib_1 & Lib_2**	5	5	0	-

Library Origin: library where the ASSET was captured from; selected ASSETs: number of randomly selected ASSETs; ASSET validation: to confirm the presence of the ASSET in the same RNA used for library construction; heteroduplex validation: amplification of alternatively spliced transcripts that could have participated in the heteroduplex formation; and cross-validation: amplification of ASSETs specifically captured from one library using the cDNA template from the other library.

### Novel alternative splicing: characterization of the putative isoforms

**Figure 5:**
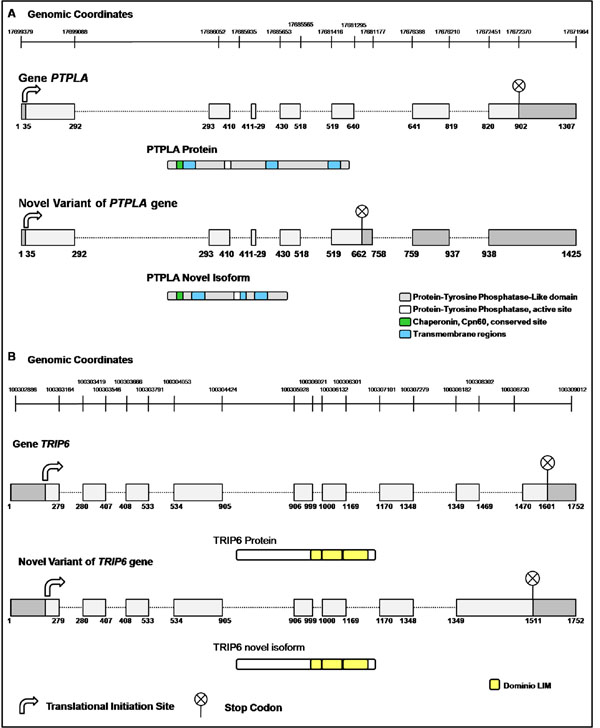
**Characterization of the novel AS variants identified.** The scheme shows the genomic structure and protein domains of the known and putative novel variants. The squares represent the exons, and the lines represent the introns. The dark regions represent the 5’and 3’ untranslated regions (UTR), the arrow represents the translational initiation site and the circles represent the stop codons. A – *PTLA* and B – *TRIP6*.

The 2 novel AS variants were characterized regarding the putative corresponding protein isoform. The *PTPLA* gene [RefSeq:NM_014241.3] codes for the member *A* of the protein tyrosine phosphatase-like family that contains proline instead of catalytic arginine. This gene contains 7 exons, and the AS variant detected in our study is due to the use of an alternative 5’ splice site of intron 5 that elongates exon 5 by 117 nt (Figure 5A). All protein functional domains found for PTPLA were also present in the novel AS detected. However, in the novel AS variant, a premature stop codon was created 96 nt upstream of the exon 5/exon 6 junction, probably leading to regulation by non-sense mediated decay (NMD) [32,33].

The *TRIP6* gene [RefSeq:NM_003302.2] is a thyroid hormone receptor interactor 6 that contains 9 exons. The novel alternatively spliced transcript reports retention of the last intron (Figure 5B). The protein coded by the *TRIP6* gene localizes to focal adhesion sites and along actin stress fibers. The novel AS variant identified also inserts a premature stop codon in the putative coding protein, without interfering with any protein functional domain.

### Evaluation of AS variant regulation by *ERBB2*-mediated expression

Finally, we investigated the putative influence of *ERBB2*-mediated expression on the regulation of AS variants for 17 ASSETS validated using *GAPDH* as a normalization factor, by comparing the expression level of the ASSETs in the C5.2 cell line in relation to the *ERBB2* basal expressed counterpart – the normal breast cell line (Hb4a) through capillary microfluidic electrophoresis (LabChip GX – Caliper Lifesciences) that accurately assesses the size and quantity of each amplification product [34].

For the 11 validated ASSETs, the relative expression levels were analyzed showing a slight influence of *ERBB2* over-expression in all ASSETs (ratio ranging from -1.9 to 1.4) (Supplemental Table 1).

**Figure 6:**
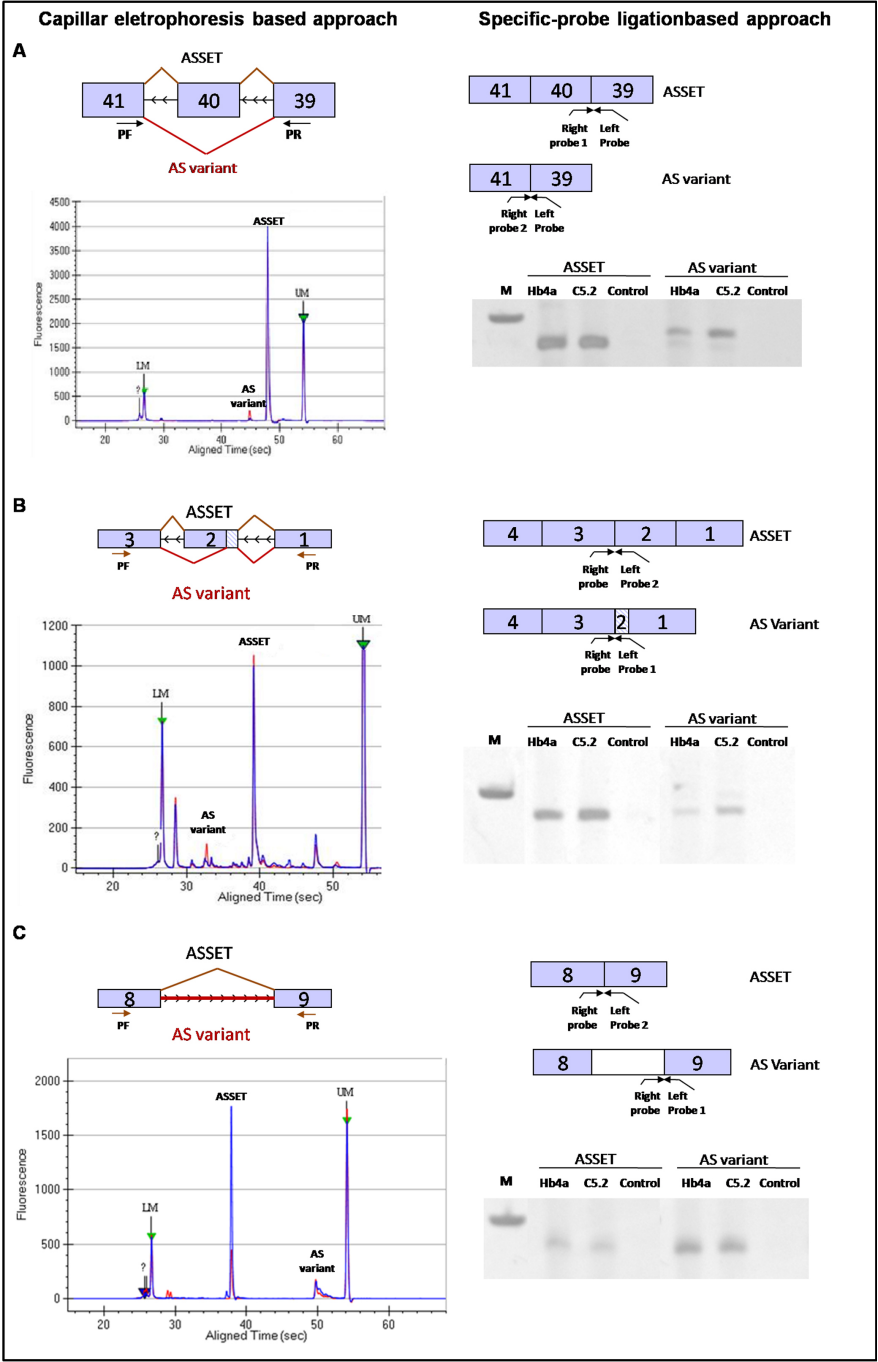
**The influence of ERBB2-mediated expression on the regulation of AS variants.** In the left panel, a schema of the microfluidic capillary electrophoresis approach is shown. The exons are represented by numbered squares according to the exons involved in the AS events for each gene. The black arrows represent the primers used for PCR amplification (PF - forward primer; PR - reverse primer). The electropherogram represents the amplification of the AS variants for the Hb4a cell line (blue line) and for the C5.2 cell line (red line). The green arrows indicates internal markers: LM (lower marker) and UM (upper marker). In the right panel, the probe-ligation approach is shown. Each pair of probe is shown for each AS variant separately, with the corresponding PCR products on 8% acrylamide gel. M - 100 bp ladder. A - gene, B - *FLNA* and C -*TRIP6*.

For the 6 ASSETS presenting an additional AS variant, the putative influence of *ERBB2* over-expression in the relative expression balance of the pairs of distinct splicing variants (ASSET and additional AS) was evaluated in both cell lines. We first calculated the expression ratio from ASSET against the variant to each cDNA template and then compared the expression ratio between the C5.2 against Hb4a cell lines. For 3 out of 6 genes, a decrease in the expression balance of the ASSET and additional AS variants was identified between the tumor and normal cell lines (Figure 6; Table 6). In more detail, the ASSETs corresponding to *SFRS9* [RefSeq: NM_003769.2] and *FLNA* [RefSeq: NM_001456.3] genes were stably expressed between cell lines, while the additional AS variants were more expressed in the C5.2 compared to Hb4a cell line (fold = 4 and 3.5, respectively) leading to a decrease of 4.84 (*SFRS9*) and 4.78 (*FLNA*) in the expression balance between the splice variants (Table 6). The ASSET of the *TRIP6* gene [RefSeq: NM_003302.2] was more expressed in the Hb4a than in the C5.2 (fold=4.6), whereas the additional AS variant presented no expression difference. These results suggested that *ERBB2*-mediated expression differently modulates the alternative splice variants of the genes *SFRS9*, *FLNA* and *TRIP6*. For the other 3 genes (*RPS2*, *PTPLA* and *ALDH3A2*), no difference in the expression balance of the AS variants between the cell lines was observed (Table 6).

**Table 6:** Gene expression analysis under the influence of ERBB2 over-expression.

Gene Symbol	Cell line	Variant	Size (bp)	Concentration (ng/ul)	Normalized Concentration	ASSET/ Variant	C5.2/Hb4a
**SFRS9**	Hb4a	ASSET	232	6.5	3.7	36.2	-4.8
		variant	100	0.2	0.1		
	C5.2	ASSET	232	6.4	2.9	7.5	
		variant	100	0.9	0.4		
**FLNA**	Hb4a	ASSET	500	24.9	14.1	95.7	-4.8
		variant	377	0.3	0.2		
	C5.2	ASSET	500	23.0	10.4	20.0	
		variant	377	1.2	0.5		
**ALDH3A2**	Hb4a	ASSET	470	20.0	11.4	10.0	1.0
		variant	610	2.0	1.1		
	C5.2	ASSET	470	24.6	11.0	10.3	
		variant	610	2.4	1.0		
**TRIP6**	Hb4a	ASSET	203	12.6	7.2	8.9	-3.4
		variant	636	1.4	0.8		
	C5.2	ASSET	203	3.5	1.6	2.6	
		variant	636	1.3	0.6		
**PTPLA**	Hb4a	ASSET	324	10.8	6.1	83.1	1.2
		variant	456	0.1	0.1		
	C5.2	ASSET	324	16.8	7.5	98.4	
		variant	456	0.2	0.1		
**RPS2**	Hb4a	ASSET	187	13.7	7.8	1.4	1.1
		variant	390	12.2	5.5		
	C5.2	ASSET	187	1.8	1.0	1.6	
		variant	390	1.4	0.6		

To confirm the alteration in the relative expression balance of AS variants mediated by *ERBB2* expression, a different approach based on probe-specific ligation and PCR amplification was applied [35]. In this strategy, 2 pairs of probes were designed for each gene, specifically targeting the variants of interest (Figure 6). The expression balance difference was confirmed for all 3 genes (*FLNA*, *SFRS9* and *TRIP6*) visualized on the acrylamide gel (Figure 6).

## Discussion

The diversity of the human transcriptional repertoire caused by AS has been extensively investigated [2,3], and it is agreed that its regulation is an important mechanism for physiological and pathological aspects of cells. Moreover, AS is a major contributor to protein diversity, which, in part, explains the high complexity of mammals compared to much simpler organisms containing a similar numbers of genes [5].

Different approaches have been used to explore the variability caused by this phenomenon, and one of the most promising strategies is the use of AS enriched cDNA libraries [25,26]. This strategy does not require previous knowledge of the variants and permits an AS transcriptome-wide analysis.

Deciphering of the human transcriptional repertoire related to AS variability is an enormous contribution in the comprehension of cancer and in the identification of more precise molecular markers in cancer.

Here we described an AS enriched cDNA library method by combining the use of trapping heteroduplex and RNA amplification procedures. The methodology was initially proposed by Watahiki and collaborators [25] and was applied in this study with some modifications to favor its application in clinically-oriented cancer studies, in which the availability of total RNA recovered from tumor tissues is normally restrictive. Moreover, the methodology established in this study is potentially applicable to RNA purified from a homogenous tumor cell population captured from a complex tissue by laser, which produces transcriptional data more correlated with the tumor cell.

Our strategy showed, in general, minimal artifacts in the identification of ASSETs, since our validation rate by RT-PCR was significantly high (94.5%). Moreover, the fact that the great majority of the AS events found in our AS enriched libraries were present in public databases and that 100% of them harbor conserved splice sites strengths the assumption that we have established a robust methodology for identifying AS in a transcriptome-wide format.

The fact that we could confirm by RT-PCR novel alternatively spliced transcripts for 2 genes to which no AS variant was present in public databases is further support that among the ASSETs with no confirmation of AS events, a high frequency of prone additional AS variants, which could participate in heteroduplex formation, is expected. The absence of amplification during the validation process of additional AS transcripts for two thirds of the selected genes suggests a significant difference in the expression level of both variants with consequent competition for the same pair of primers in the PCR reaction, avoiding the amplification of low-abundance AS transcripts.

The relatively high redundancy levels encountered in both libraries (84.78% and 86.84%) were somewhat expected. This number is similar to the redundancy reported by Thill and collaborators [26]. In technical terms, this problem can be bypassed by decreasing the number of PCR cycles in the library construction, which is relatively easy to control.

Another potential problem was that no additional alternatively spliced transcripts were identified in sequence data provided by enriched cDNA libraries alone. This can be indicative of a problem caused by using non-phosphorylated adaptors. In this situation, only one strand (5’-3’) of these adaptors was ligated to the 5’ end of the *Dpn*II digested molecules that contains a phosphate residue; the other strand (3’-5’) was not ligated and, as a consequence, was disconnected from the cDNA molecules at the denaturation step of PCR and was consequently unable to be cloned and sequenced. Usually this region is re-synthesized by polymerase at the first cycle of the PCR reaction through annealing of complementary regions of cDNA molecules, a process known as polymerase fill-in, also seen in some cDNA library approaches [36,37]. However, in our case where the strands of cDNA molecules are from distinct alternatively spliced transcripts, the fill-in process is probably inefficient due to non-perfect annealing. To avoid this problem, the use of phosphorylated adaptors is a simple solution that would favor the representation of both alternatively spliced transcripts that formed the heteroduplex structure.

*ERBB2*, or *HER-2/neu*, is an oncogene that is over-expressed in 20-30% of human breast carcinomas and is associated with poor prognosis, independent of the lymph node status [38,39]. This marker is also associated with chemo resistance to a range of anticancer drugs and a positive response to herceptin [40,41]. Despite this oncogene being most extensively investigated in clinical and basic oncology, the *ERBB2*-mediated mechanism involved in the transformation and progression of breast tumors has not yet been totally elucidated.

In this study, we proposed to identify alternatively spliced transcripts associated with breast tumors that are under *ERBB2* influence by constructing 2 AS-enriched cDNA libraries using RNA sources representative of *ERBB2* over-expression: the human breast cell line C5.2 that was previously transfected with 4 copies of full-length *ERBB2*[42] and a pool of 5 breast carcinoma samples, which demonstrate strong positivity in ERBB2 immunostaining in tumor cell membranes [43].

For testing if the expression of ASSETs was regulated by *ERBB2*-mediated expression, we evaluated the ASSETs validated by RT-PCR in both cell lines, HB4a and C5.2, the former with basal levels and the latter with over-expression of *ERBB2* mRNA [44]. Both cell lines have been considered a model for the investigation of *ERBB2*-mediated expression, since the only difference between them is the insertion of 4 copies of full-length *ERBB2* in the C5.2 cell line [45,46]. For the ASSETs in which we could identify an additional AS transcript by RT-PCR, 50% of them (3 out of 6 - *TRIP6*, *FLNA* and *SFRS9*) seemed to be influenced by *ERBB2*-mediated expression, since differences in the relative expression balance between both cell lines were observed.

Although the expression assessment of 2 or more AS variants is a problematic issue concerning accurate quantification the results presented here were confirmed by an alternative methodology, which increased the robustness of the data.

The microfluidic capillary electrophoresis-based strategy relies on amplification of both variants in the same reaction and could introduce bias due to amplification competition between variants. However, this would be expected to equally influence all reactions, independent of the template used. The alternative strategy relies on the specific binding of probes under highly stringent conditions, enabling the evaluation of each variant separately, with high accuracy and is consequently very promising for AS expression assessment. The different expression balance between both cell lines for 3 genes confirmed by 2 different approaches suggests that these genes transcribe AS variants, whose expression is differently influenced by *ERBB2*.

*FLNA* is a member of the actin-binding protein family that organizes actin filaments and is involved in numerous cellular processes, especially development. Many studies have reported the involvement of this protein in carcinogenesis. Using melanoma cell lines lacking or expressing *FLNA*, Fiori and collaborators [47] have shown that this protein is an important regulator of EGFR members (including *ERBB2*) that ensure efficient ligand-mediated activation of these receptors and, consequently, intracellular trafficking and degradation.

*SFRS9* is a RNA-binding protein from the arginine/serine-rich family that acts as a splicing factor regulating constitutive splicing and also modulating the selection of alternative splice sites. It has been suggested that this protein acts downstream of the ERBB2 pathway, since phosphorylation of SFRS9 was detected in *ERBB2*-over expressing breast and ovarian cancer cells and was reduced by monoclonal antibody *Herceptin* treatment. Moreover, a putative role for SFRS9 in cell migration was suggested, since migration was significantly retarded following the depletion of *SFRS9* transcripts in ovarian cancer cell lines [48].

TRIP6 is a thyroid hormone receptor interactor that localizes to focal adhesion sites and along actin stress fibers [49,50]. This protein enhances lysophosphatidic acid (LPA) -induced cell migration by directly binding to the carboxyl-terminal tail of the LPA2 receptor through its LIM domains [51]. Moreover, TRIP6 might enhance cell migration by binding to PDZ domain of MAGI-1b/PTEN destabilizing membrane β-catenin and E-cadherin junctional complexes, promoting cell motility [52].

The development of strategies to selectively represent the AS transcripts repertoire, requiring small amounts of total RNA, will be important for generating more correlated information between AS transcripts and specific cell types and conditions in a transcriptome-wide format.

In spite of using Sanger sequencing in this study, our approach is completely suitable for using with next-generation sequencers [53], with the possibility of decreasing the number of PCR cycles, and consequently the redundancy level of the library; and assaying multiple barcoded samples with high sequence coverage in a single run. Finally, the use of next generation sequencers would tremendously expand the applicability of our approach toward characterizing cancer cell transcriptome diversity resulting from AS.

## Conclusions

In this study we presented a method for exploring AS from any RNA source that generates reliable AS data in a transcriptome-wide format. Additionally, our data identified AS transcript candidates whose expression was influenced by *ERBB2*-mediated expression and can be tested as molecular markers for breast cancer. The association of such methodology with deep sequencing may be helpful for completely deciphering the cancer cell transcriptome and finding more precise molecular markers.

## Methods

### Samples

The human breast cell line C5.2 is derived from normal luminal cells transfected with four copies of the full-length ERBB2 cDNA (HER-2/neu) gene presenting tumor characteristics [42]. Cells were maintained in RPMI medium supplemented with 100 ml/l fetal bovine serum (FBS), 5 µg/ml insulin, 5 µg/ml hydrocortisone and 1 mmol/l L-glutamine in a humidified incubator containing 50 ml/l CO_2_ at 37°C. The medium was changed every 2-3 days, and after 10 days the total RNA was extracted using a CsCl gradient [54]. The yield of extracted total RNA was determined with a Kontron 810 spectrophotometer GeneQuant pro (GE Healthcare Life Sciences), and the integrity was also verified by electrophoresis through 1% agarose gel upon visualization with ethidium bromide.

RNA samples from 5 ductal breast carcinoma samples used in this study were provided by the biorepository bank from A.C. Camargo Hospital (São Paulo, Brazil). These samples were positive for ERBB2 through immunohistochemistry analysis (Table 6), according to the following criteria: weak to moderate complete membrane staining in > 10% of tumor cells or strong complete membrane staining in > 30% of tumor cells.

### Alternative splicing enriched cDNA library construction

### RNA amplification and double strand cDNA synthesis

For first strand cDNA synthesis, total RNA was incubated with 0.75 µg oligo dT containing the T7 RNA polymerase site (5’AAACGACGGCCAGTGAATTGTAATACGACTCACTATAGGCGCT(24)’3’) at 70°C for 10 minutes. The reaction was performed by adding 1X first strand buffer, 0.01 M DTT (Dithiothreitol), 40 U of RNasin (Promega Corporation), 1 mM dNTP (GE Healthcare Life Sciences), 1 µg of Template Switch (TS) DNA Oligo (5’AAGCAGTGGTAACAACGCAGAGTACGCGGG 3’) and 400 U of SuperScript II (Invitrogen Life Technologies) in a total volume of 20 µl. The reaction was incubated for 120 min at 42°C. For the second strand synthesis, the Advantage® cDNA PCR Kit (Clontech) was used as follows: 5X cDNA PCR Reaction Buffer, 1 mM dNTP Mix, 5X Advantage cDNA Polymerase Mix, 1.4 U of RNase H (Invitrogen Life Technologies) in a final volume of 100 µl. The reaction was incubated at 37°C for 10 min, 94°C for 3 min, 65°C for 5 min. and 75°C for 30 min. The stop reaction including 0.25 M of NaOH and 0.5 mM EDTA was added, followed by incubation at 65°C for 10 min. The dscDNA was purified by phenol:chloroform:isoamylic alcohol (25:24:1) pH 8.0 extraction followed by Microcon YM-100 Centrifugal Filter Unit (Millipore).

Double-strand cDNA was in vitro transcribed into RNA with RiboMAX^™^ Large Scale RNA Production Systems (Promega Corporation) as follows: 1X buffer, 3 µM rNTP and 2.5 µl Enzyme T7 Mix. The reaction was incubated at 37°C for 6 hours. Amplified RNA (aRNA) was purified by TRIzol® Reagent (Sigma Aldrich Corporation).

After purification, aRNA was used for double-stranded cDNA synthesis as described above using 1 µg of TS-oligo for the first strand synthesis and 0.5 µg oligo dT(24) for the second strand synthesis.

### Denaturation and renaturation

Double-stranded cDNA molecules were heated at 96°C for 20 min and incubated at 42°C for 24 hours in a mixture of 0.2% SDS, 0.5 M NaCl, 0.05 M Tris-HCl pH 7.5 and 30% formamide.

### Exonuclease VII cleavage

Exonuclease VII (USB Corporation) cleavage was performed in 70 mM Tris-HCI, pH 8.0; 8 mM EDTA, pH 8.0; 10 mM 2-mercaptoethanol; 50 µg/ml BSA and 0.2 U of the enzyme and incubated at 37°C for 30 min. The enzyme was inactivated at 95°C for 10 min.

### *Dpn*II digestion

Fifteen units of the restriction enzyme II (New England Biolabs) was used for each 500 ng of cDNA in 1X buffer. The reaction was incubated at 37°C for 3 hours.

### Heteroduplex molecule trapping by biotin-streptavidin

The cDNA sample was incubated with 100 pmoles of random 25-mer oligonucleotide biotinilated at the 5’ end in 6X SSC and 0.1% SDS at 65 °C for 16 hours.

This mixture was incubated with 1 mg streptavidin magnetic particles (F. Hoffmann-La Roche Ltd.) and 300 μl TEN100 binding buffer (10 mM Tris-HCl; 1 mM EDTA; 100 mM NaCl, pH 7.5) for 30 min at room temperature. The tube was applied to a magnetic separator, and the supernatant was removed and incubated with another aliquot of streptavidin magnetic particle for a second round of purification. Both aliquots of magnetic particles coupled to heteroduplex molecules by the biotinilated random oligonucleotide were mixed and washed 3 times with TEN100 washing buffer (10 mM Tris-HCl; 1 mM EDTA; 1 M NaCl, pH 7.5). The cDNA molecules were then eluted from the magnetic particles by adding 6 M guanidine-HCl and purified by a phenol: chloroform: isoamylic alcohol pH 8.0 extraction.

### Ligation of XDPN12 and XDPN14 adaptors

The adaptors were commercially synthesized and contained four bases complementary to the cleavage site of the *Dpn*II enzyme. First, the cDNA molecules were mixed with 1X T4 Ligase Buffer, 400 pmols XDPN12 (5’GATCTCTCGAGT3’) and 400 pmols XDPN14 (5’CTGATCACTCGAGA3’) and incubated at 55°C for 1 min. Next, the temperature was decreased from 54°C to 28°C at a rate of 2°C every 2 min and from 28°C to 14°C at a rate of 2°C every 4 min to favor a perfect annealing of the oligonucleotides. At last, 2000 units of T4 DNA ligase (Invitrogen Life Technologies) were added, and the reaction was incubated at 14°C for 16 hours. The reaction was purified with a Microcon YM-100 Centrifugal Filter Unit (Millipore).

### Polymerase chain reaction

The RT-PCR reaction was carried out in 1X buffer, 0.1 mM dNTP, 1.5 mM MgCl2, 200 pmols XDPN18 oligonucleotide (5’CTGATCACTCGAGAGATC3’), 2 units GoTaq® DNA Polymerase (Promega Corporation) and 10 µl of purified cDNA in a total volume of 20 µl. The reaction was incubated at 95°C for 4 min followed by 35 cycles of 95°C for 45 s, 58°C for 1 min and 72°C for 4 min and a final extension at 72° for 7 min.

### Cloning and sequencing

PCR products were inserted into T/A plasmid vector pTZ57R/T using the InsT/Aclone PCR Product Cloning Kit (Fermentas Life Sciences), following the manufacturer’s recommendations, in a total volume of 10 µl. The ligation was performed at 22°C for 16 hours. The ligation was dialyzed for 20 min in 0.025 µM nitrocellulose membrane (Millipore), and 3 µl was used for transformation in DH10B E. coli cells by electroporation (2.5 KV, 25 μFD, 200 OHMS). The clone inserts were sequenced with ABI Prism 3100 (Applied Biosystems). The sequencing reaction was performed with M13 reverse primer (5’GTCATAGCTGTTTCCTG3’) and BigDye Terminator v3.1 cycle sequencing kit (Applied Biosystems), following the manufacturer’s recommendations.

### Bioinformatics analysis

The sequences were automatically analyzed, and regions corresponding to vector sequences were trimmed. The quality control was performed in 20 bp windows, where only windows containing at least 15 bp with a Phrep quality score ≥ 20 were considered.

The sequences of each library were clustered individually using the CAP3 program, allowing estimation of library’s redundancy. The consensus sequences were first aligned against the human genome (NCBI build #36.1) using BLAT [29]. Second, to improve the quality and specificity of alignment the best hit of each sequence in the genome was selected, and realigned using Sim4 [30]. Third, sequences showing identity ≥ 93% and sequence coverage (percentage of sequence length aligned) ≥ 55% were considered. Lastly, the sequences were clustered with ESTs from dbEST (8,133,299 sequences), mRNAs (244,284 sequences) and RefSeqs (26,040 sequences) downloaded from UCSC (September 2007) (see Galante [55] for more details).

### RT-PCR validation

The primers for splice variant validation were designed at the extremities of the ASSET sequence. Twenty nanograms of cDNA from both the total RNA from the C5.2 cell line and the pool of breast cancer samples were used to validate the ASSETs from Lib_1 and Lib_2, respectively. The PCR reaction was performed in a total volume of 20 μl by mixing 1 X reaction buffer (Invitrogen Life Technologies), 2.5 mM MgCl_2_ (Invitrogen Life Technologies), 0.2 mM dNTP (Amersham Biosciences), 10 pmoles of each primer and 1 unit Taq Platinum (Invitrogen Life Technologies). PCR reactions were performed with 40 cycles at 95°C for 30 sec, 60°C for 30 sec and 72°C for 30 sec, followed by a final extension at 72°C for 7 min. Amplification products were visualized on a 8% acrylamide gel and subsequently sequenced by ABI3130.

### *ERBB2* influence on relative expression

For verifying the *ERBB2* influence on gene expression, all ASSETs were amplified using the C5.2 cell line and also the Hb4a cell line, which is a human mammary luminal epithelial cell line. The PCR products were quantified through capillary microfluidic electrophoresis (LabChip GX – Caliper Lifesciences). The expression of the *GAPDH* gene was used as a normalization factor. The expression ratio was determined by the normalized value of C5.2 divided by the normalized value of Hb4a for each ASSET. Genes were considered to be differently expressed between cell lines for ratios ≥|2|. The differently expressed genes were analyzed in a group of tumor and normal breast samples through a strategy based on specific-probe ligation. The left and right probes were targeted against specific exon junctions of each variant of a gene. The left probe contained at its 5’ end a recognition sequence of the forward PCR primer (GGGTAGGCTAAGGGTAGGA) followed by a stuffer sequence of 38 nucleotides (CCGTTGCCAGTCTGCTCAGACCTCCCTCGCGCCATCAG), and the right probe was phosphorylated at its 5’ end and contained a recognition sequence of the reverse PCR primer (TCTAGATTGGATCTTGCTGGCAC) at its 3’ end. A specific RT primer designed downstream of the probe target sequence was used for cDNA synthesis. The probes were hybridized to pre-heated cDNA from Hb4a and C5.2 at 60°C overnight, and only the probes specifically hybridized to their target sequences were connected by T4 DNA ligase, resulting in one unique probe. As a negative control, ligation and hybridization were performed in the absence of any template for all pairs of probes. The unique probes were PCR amplified. Amplification products were analyzed on 8% acrylamide gel. (Additional file 1)

### List of abbreviations used

AS: alternative splicing; ASSET: alternative splicing sequence-enriched tag; dscDNA: double-stranded cDNA; EST; expressed sequence tag; RT-PCR: reverse transcriptase polymerase chain reaction; TS-oligo: Template Switch oligo

## Competing interests

The authors declare that they have no competing interests.

## Authors' contributions

ENF designed the study, carried out all wet lab assays and wrote the manuscript. MCRR participated in the design of the study and helped with the construction of the libraries. PAG and JES performed the bioinformatics analysis. GCM participated in the validation experiments. SJS conceived the study and coordinated the bioinformatics analysis. DMC conceived, designed and coordinated the study and wrote the manuscript.

## Supplementary Material

Additional File 1 Gene expression analysis under the influence of *ERBB2* over-expression. Click here for file
